# The Value of Learning about Natural History in Biodiversity Markets

**DOI:** 10.1371/journal.pone.0144047

**Published:** 2015-12-16

**Authors:** Douglas J. Bruggeman

**Affiliations:** Ecological Services and Markets, Inc., Marshall, NC 28753, United States of America; University of Waikato (National Institute of Water and Atmospheric Research), NEW ZEALAND

## Abstract

Markets for biodiversity have generated much controversy because of the often unstated and untested assumptions included in transactions rules. Simple trading rules are favored to reduce transaction costs, but others have argued that this leads to markets that favor development and erode biodiversity. Here, I describe how embracing complexity and uncertainty within a tradable credit system for the Red-cockaded Woodpecker (*Picoides borealis)* creates opportunities to achieve financial and conservation goals simultaneously. Reversing the effects of habitat fragmentation is one of the main reasons for developing markets. I include uncertainty in habitat fragmentation effects by evaluating market transactions using five alternative dispersal models that were able to approximate observed patterns of occupancy and movement. Further, because dispersal habitat is often not included in market transactions, I contrast how changes in breeding versus dispersal habitat affect credit values. I use an individually-based, spatially-explicit population model for the Red-cockaded Woodpecker (*Picoides borealis)* to predict spatial- and temporal- influences of landscape change on species occurrence and genetic diversity. Results indicated that the probability of no net loss of abundance and genetic diversity responded differently to the transient dynamics in breeding and dispersal habitat. Trades that do not violate the abundance cap may simultaneously violate the cap for the erosion of genetic diversity. To highlight how economic incentives may help reduce uncertainty, I demonstrate tradeoffs between the value of tradable credits and the value of information needed to predict the influence of habitat trades on population viability. For the trade with the greatest uncertainty regarding the change in habitat fragmentation, I estimate that the value of using 13-years of data to reduce uncertainty in dispersal behaviors is $6.2 million. Future guidance for biodiversity markets should at least encourage the use of spatially- and temporally-explicit techniques that include population genetic estimates and the influence of uncertainty.

## Introduction

Use of tradable credit systems to mitigate impacts of development on biodiversity has become increasingly common [1] despite the lack of evidence that such programs achieve conservation goals [2–5]. These programs assume that purchase of a credit, which represents an increase in ecological quality at one location, is sufficient to offset the loss of an ecological resource to development at another location. The increasing popularity of tradable credit systems may reflect a stronger fit of policies to traits of *Homo sapiens*, which may have flourished thanks to trade [6], than to the traits of the species they attempt to protect.

Even if a habitat trade is able to offset the same habitat area and quality, changes in the spatial configuration of habitat, often referred to as habitat fragmentation or conversely habitat connectivity, occur. Levels of habitat fragmentation can have significant impacts on population viability [7–9], however we often lack the detailed natural history data required to understand habitat fragmentation effects. In the U.S., the best available science is required to evaluate mitigation options [10, 11], which effectively means that uncertainty can not be used to prevent a trade. If knowledge of habitat fragmentation effects are unknown (i.e., “not available”) or if land cover types that facilitate movement (i.e., dispersal habitat) have not been identified, landscape considerations could be excluded when determining equivalency of trades. Precautionary approaches in which expert opinion is used to account for a range of uncertain landscape-scale effects within trades could be employed, but the ability of precautionary approaches to adequately make tradeoffs between habitat area and connectivity in a cost-effective manner has not yet been demonstrated in the field.

Current mitigation practice does a poor job of including the impacts of habitat fragmentation into credit values. Credit values are often based on habitat area and/or abundance [2]. As landscapes are fragmented, wildlife populations become subdivided in individual patches. Consequently, the smaller population size and increased isolation leads to increased risks of local extinction as well as rates of genetic drift and inbreeding [12]. Habitat area and abundance are not sensitive indicators of the effect that habitat fragmentation has on rates of migration, genetic drift, inbreeding, and local extinction. Rather, recent work has shown that population genetic data are a more sensitive indicator of fragmentation effects [9, 13, 14]. The advent of digital mapping has led to applying geometric summaries of landscape structure, or landscape indices, to trades [15]. However, landscape indices have not been shown to be predictive of habitat occupancy in landscapes in which patterns change over time [16, 17].

To evaluate tradeoffs between complexity and accuracy, Bruggeman and Jones [15] contrasted conservation value of habitat trades by applying both landscape indices and an individually-based, spatially-explicit population model (IB-SEPM) [18], which have a proven ability to predict biological patterns in dynamic landscapes [19, 20]. Two alternative trades were considered, one that met the precautionary goals specified under the recovery plan [21] for habitat area and connectivity (estimated with functional landscape indices) and a second that substituted habitat connectivity for habitat area, leading to a net decrease in habitat area. Total population persistence increased under both trades but more so when connectivity was substituted for habitat area. Importantly, for the trade meeting the recovery plan, rates of local extinction increased for many patches across the landscape, but a similar increase in rates of local extinction was not observed for habitat patches when connectivity was substituted for habitat area.

Such an increase in rates of local extinction is a network externality [22], or a secondary consequence that results from a change in a spatially-dependent system. While such consequences of spatial-dependence are not surprising for many ecologists, network externalities could be ignored when using simple trading rules based on landscape indices rather than an IB-SEPM. Further, these results indicate that our ability to incorporate adequate precautions against network externalities may be limited when using simple trading rules based on landscape indices, which may underestimate the landscape-scale effects of a take. We lack guidance regarding how to make tradeoffs between local and regional effects of landscape change for species [13]. Recognizing possible changes in local extinction is critical for landowners working to either preserve or trade habitat in a cost-effective way.

Economists evaluating subsidy payments for biodiversity have also included the influence of spatial-dependence. While these studies demonstrate how to relate payments to spatially-dependent biological processes under imperfect data on costs [23, 24], facilitate cooperation to achieve specific habitat geometries [22], and derive auction conditions so that landowners reveal true costs [24], the studies are limited by the use of simple assumptions regarding how spatial patterns affect biodiversity. The simplifying assumptions do facilitate fitting biological dynamics into rigorous economic analysis. In reality, the spatial pattern of habitat that minimizes negative network externalities will vary by species [12, 25]. Populations persist at broad spatial scales through interplay of reproduction within habitat patches and migration among habitat patches [26]. Population genetic theory indicates that population viability can often be enhanced by a balance between connectivity and isolation [12, 13]. Therefore, the best habitat pattern will depend on the interplay between migration, genetic drift, and local extinction—processes that change along with landscape structure. Therefore, such conservation questions should also consider the influence of the timing of landscape change, or temporal heterogeneity, on the determination of the best spatial pattern [12, 13]. The interaction between temporal-, spatial-feedback mechanisms have often been ignored in conservation [24, 27–30] but are now receiving more attention [31].

To balance the regional and local effects while including temporal effects of a mitigation market, Bruggeman at al [12, 13] derived Landscape Equivalency Analysis (LEA). LEA is an extension of the resource-based compensation approach [32] that integrates cap and trade approaches in environmental economics with evolutionary theory to prevent trades from exacerbating habitat fragmentation effects [12, 13]. In a traditional tradable credit system for industrial air emissions the externality is air pollution and firms able to reduce pollution at a lower cost sell credits to firms with higher abatement costs [33]. In contrast, under a mitigation banking market a positive network externality results when a mitigation bank reduces habitat fragmentation effects, and when habitat patches are lost to development, as trading commences, any increase in negative network externality can not be greater than the positive network externality provided by the bank [12, 13]. LEA accomplishes this by placing a “cap” on fragmentation effects by preventing trades that move the spatial allocation of genetic diversity farther away from the allocation observed in a “pre-settlement landscape” [12, 13].

Past research illustrated how LEA captures changes in the balance between recruitment and migration that result due to habitat trading using population genetic indices [13]. The conservation value of a LEA credit increased as habitat trades move the balance between rates of recruitment and migration closer to levels observed in the pre-settlement apportionment of habitat. This increase in conservation value reduces the probability that changes in recruitment and migration would lead to the expression of deleterious traits that result from mating between close relatives (inbreeding depression). Similarly, this reduces the probability that changes in migration would disrupt locally adapted gene complexes due to mating between individuals from different regions (outbreeding depression). In this way, LEA focuses on protecting the environmental context that permitted adaptive evolution, rather than attempting to identify all adaptive components of genetic variance [34, 35]. LEA also allows managers to identify a large number of different patterns of habitat that make equivalent contributions to population viability. Importantly, no empirical genetic data are required to apply LEA, as described in this study, but such data can be used if available.

However, LEA does not solve the problem of estimating how habitat fragmentation affects populations. LEA so far has relied on IB-SEPMs that include both demographic and genetic components of population structure. IB-SEPMs were initially criticized for containing large uncertainties due to the amount of data required to parameterize models of complex systems [36, 37]. However, techniques in inverse-modelling have been derived to provide an explicit approach for reducing uncertainty and indicate that IB-SEPMs can be constructed and validated with less data than previously imagined [38]. Bruggeman et al. [39] applied inverse modeling to estimate dispersal parameters for an IB-SEPM for the Red-cockaded Woodpecker (RCW; *Picoides borealis*). Information theory was applied to find the set of dispersal parameters that most faithfully reproduced patterns of movement and habitat occupancy observed in the field.

This study extends the results of Bruggeman et al [39] to estimate the value of reducing uncertainty in dispersal parameters, a critical component underlying habitat fragmentation effects, within a tradable credit system. Decision analysis is used to determine the expected value of removing uncertainty, thereby providing a way to determine if further data collection would be cost-effective [40, 41]. Information may have very high value in these biodiversity markets as acquiring knowledge regarding how landscape change affects reproduction and migration could lead to markets able to provide greater certainty of population persistence at a lower cost. Therefore, while uncertainty cannot be used to prevent trading, there may be tradeoffs between the value of tradable credits and the value of information that reduce uncertainty in the value of credits.

Here, I apply the five dispersal models identified using inverse modeling [39] to habitat trading scenarios—thus, providing a novel basis for applying decision analysis to tradable credit systems so that changes in network externalities under uncertainty can be estimated. I also included a sixth dispersal model that represents the state of knowledge prior to applying inverse modeling [42]. The techniques included in this paper have been applied in real landscapes [43], but my objective here is to use hypothetical trades to highlight more general implications of habitat trading and network externalities. I simulated eight landscape treatments, which could be considered different trades (Table 1). The trades vary the temporal changes in breeding and dispersal (or matrix) habitat to contrast the roll of transient dynamics in breeding versus dispersal habitat may have on credit values. Then, given current uncertainty in dispersal behaviors, I estimate the value of reducing uncertainty regarding dispersal for mitigation markets.

**Table 1:** Description of how habitat loss was applied across landscape treatments after the addition of the bank in year 25. Two areas were considered as takes, “A” and “B” (Fig 1).

Take Landscape Treatment	Take of high quality habitat	Matrix habitat
A	A at yr 40	static
B	B at yr 40	static
AB5	A at yr 40, B at yr 45	static
BA5	B at yr 40, A at yr 45	static
AB20	A at yr 40, B at yr 60	static
BA20	B at yr 40, A at yr 60	static
Amatrix	A at yr 40	1% loss for 40 yrs starting at yr 26
Bmatrix	B at yr 40	1% loss for 40 yrs starting at yr 26

## Methods

### Individually-based, spatially-explicit population model

The RCW IB-SEPM simulates the cooperative breeding system of RCWs, and was constructed based on a 15-year banding program for RCWs [44]. Breeding groups consist of male and female breeders, fledglings, and, helpers who are usually male and full or half-sibs to the fledglings [45]. Male helpers play a critical role in population dynamics by participating in the defense of the territories, feeding of nestlings, and inheriting their natal territory upon the death of the male breeder. Male helpers will preferentially inherit their natal territory upon the death of the breeding male, out-competing floaters and helpers in adjacent territories. Floaters of both sexes are also present in the region, which move continuously seeking a breeding vacancy in a territory [45]. The fate and behavior of every individual is tracked within the model using a seasonal time-step and a spatial grain of 100 m x 100 m across a 547,600 ha landscape. Details of the RCW IB-SEPM can be found in the S1 Appendix following the Overview, Design concepts, and Details protocol, which is intended to provide a standard approach for describing individual-based models across studies to increase transparency [46].

### Dispersal uncertainty

Recent research has revealed much complexity in RCW dispersal, in which dispersal behaviors are affected by a combination of life history stage, dispersal mode, and landscape composition [39, 47, 48]. For example, radio-telemetry revealed that juvenile females respond differently to forest structure whether the birds are embarking on short distance or long distance dispersal [48]. During short distance movements birds prefer to disperse through forested areas and perceive barriers as areas with minimal forest cover >8 m tall [38].

In order to estimate behaviors across multiple life stages and dispersal modes in a one-million hectare landscape, Bruggeman et al. [39] applied Pattern Oriented Modeling (POM) [38], a form of inverse modeling, to estimate dispersal parameters for the RCW IB-SEPM. Alternative dispersal models were assembled by identifying ranges for seven parameter values used to simulate dispersal based on previous studies and expert opinion (S1 Appendix). Values from these ranges were sampled randomly with replacement using a Latin hypercube until 600,000 alternative models were assembled. Results of inverse-modeling confirmed the preference for forested areas during movement, but also indicated uncertainty regarding the strength of the barrier effect [39]. To limit dimensionality, the five models best able to approximate observed patterns of abundance and connectivity were used to evaluate habitat trades. To provide a comparison with knowledge of dispersal behaviors used in decision making prior to applying POM, the dispersal model used in the RCW Decision Support System (DSS) [49] was also applied, referred to as model 6(DSS).

### Simulating trades using alternative landscape treatments

The conservation value associated with alternative habitat trades was estimated by applying the IB-SEPM to different landscape treatments. The landscape treatments represent different configurations of land cover types. The landscapes included land cover classified as high quality habitat (mimicking Longleaf pine savannah that serves as breeding and foraging habitat [50]), matrix habitat mostly used for movement (i.e., mixed pine or hardwood forests), and non-forested areas, which may serve as barriers during movement [39, 48]. These land cover classes were assembled in different ways to simulate a conservation banking scenario in which a bank is added to the landscape, generating credits, and then “takes”, or loss of habitat, occur that later times, generating debits. In order to create realistic spatial associations of land cover types, in which high quality habitat is surrounded by matrix habitat and matrix habitat is surrounded by non-forested areas, maps were created by using a three-dimensional surface [51].

**Fig 1:**
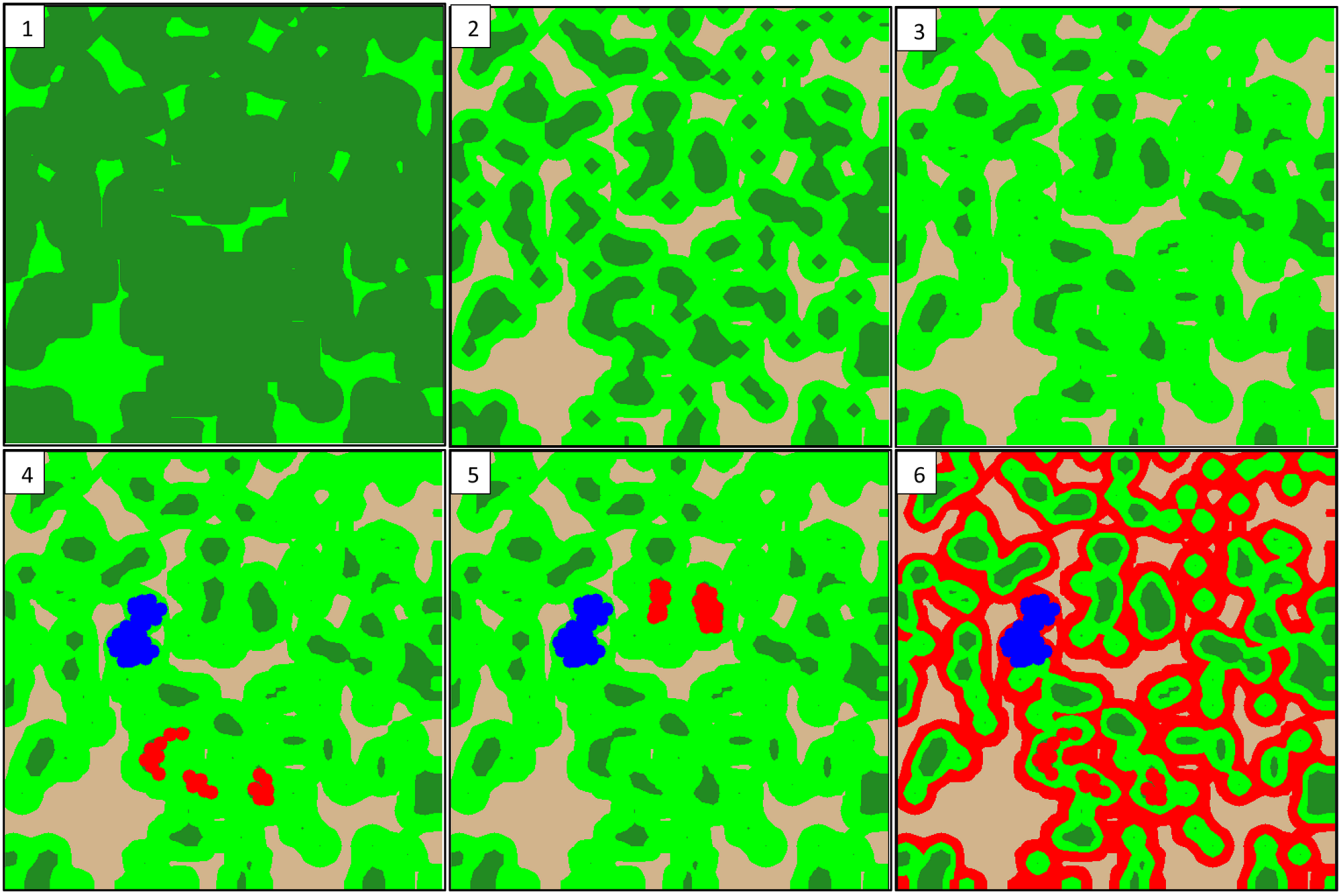
Landscape treatments used to estimate the value of tradable credits at a landscape-scale. 1) Pre-settlement landscape, with 85% high quality habitat (dark green) and 15% matrix habitat (light green); 1) Recovery landscape, with 25% high quality habitat, 55% matrix habitat, and 20% non-forested (gray); 3) Baseline landscape, with 10% high quality, 70% matrix habitat, and 20% non-forested; 4) Bank and Take A landscape, with addition of bank at year 25 as high quality habitat represented in blue and loss of habitat in area A represented in red (take treatment A); 5) Bank and Take B landscape (take treatment B); and 6) Bank and Take A landscape with total matrix habitat lost by year 40 in declining matrix treatment (take treatment Amatrix). Take treatments AB5, BA5, AB20, BA20, Bmatrix are not shown but can be inferred from existing figures.

For a baseline landscape, or a fragmented landscape inhabited by the at-risk species at the start of this hypothetical market, two horizontal planes were placed within the three-dimensional surface at two elevations, producing a map with three different land cover classes. The baseline landscape treatment contained 10% high quality habitat, 70% matrix habitat, and 20% non-forested (Fig 1). Past studies used a baseline landscape with 3% high quality habitat to examine the interplay between regional extinction, local extinction, and the erosion of genetic diversity [13, 15]. However, for this study the baseline landscape begins with 10% high quality habitat to demonstrate tradable credits in a landscape without the influence of regional extinction.

Other landscape types were also considered to construct a conservation banking scenario, in which I assumed habitat is restored near the center of the landscape, creating a bank. The bank landscape treatment, or the baseline landscape with the addition of a conservation bank, was created by assuming matrix habitat was restored at year 25 in an area on the map that was high-quality habitat in the pre-settlement treatment, creating space for 25 territories. Here I assume artificial cavities are created and made available in year 25 and birds colonize new territories on their own without translocation [13].

Then, the removal of habitat by landowners seeking to develop their land (i.e., incidental take) is simulated by changing the classification of habitat patches to “non-forested”. To simulate a habitat trade, eight alternative take landscape treatments were considered, each simulating the loss of habitat in different ways (Table 1). The loss of high quality habitat in year 40 was simulated at two different areas, “A” and “B” (Fig 1), each containing 14 RCW territories—thus allowing 15 years of natural colonization of the bank prior to habitat loss. The loss of these areas was also considered individually and in series, separated by five or 20 years, providing four landscape treatments (Table 1). The last two landscape treatments included the incremental loss of matrix habitat over many years. For these declining matrix treatments, starting in year 26 and continuing for another 39 years, a one percent loss in matrix habitat was implemented for the baseline, bank, and take landscapes by shifting one of the horizontal planes applied to the three dimensional surface each year, until non-forested areas increased from 20% to 60% and matrix habitat was reduced from 70% to 30% of the landscape (Fig 1).

In order to provide reference landscapes used by LEA to estimate tradable credits, two other landscapes were included. A pre-settlement landscape was constructed to represent the historic spatial distribution of habitat. The pre-settlement map contained 85% high quality habitat with the remainder being hardwood forest (i.e., matrix habitat). The final landscape treatment was constructed to represent a recovery landscape, or a landscape fragmented in a similar manner to the baseline landscape but with greater habitat area allowing for a demographically stable population. The assumption here is that, as in the field [50], some matrix habitat in the baseline landscape could be restored to breeding and foraging habitat quality, thus increasing patch size (Fig 1).

### Estimating network externalities

LEA ensures that habitat patches traded make at least equivalent contributions to rates of recruitment and migration estimated at a landscape-scale. For this analysis I assume the abundance and genetic diversity of the endangered species provide benefits to the public, or “services” [12, 52, 53]. A habitat patch lost is considered equivalent to a habitat patch gained if there is no change in services at a landscape-scale due to the trade. By using population genetic theory rather than summaries of habitat geometry, we can capture changes in genetic drift, inbreeding, migration, and local extinction that occur in non-equilibrium landscapes [12, 13]. Ideally, if we understood how changes in genetic diversity affected population growth then we could avoid estimating separate credits for genetic diversity [12].

The change in spatial apportionment of genetic variation is based on Nei’s theory genetic diversity in subdivided populations, which relates total genetic diversity (H_T_) to the average genetic diversity within breeding groups (H_S_) and average genetic divergence among breeding groups (D_ST_), *H*_*T*_ = *H*_*S*_+*D*_*ST*_. Due to the monogamous mating system of RCWs, breeding groups were treated as the “subpopulation” because they represent the spatial scale at which alleles are combining at random [54]. To include genetics in the simulation, every adult bird seeded at the start of the simulation is considered a founder (S1 Appendix) and was assumed to be unrelated and contained one heterozygous locus with two unique alleles (i.e., total alleles = 2 x number of breeders in the founding population, an Infinite Alleles Model [IAM] of genetic variation). Therefore, as described in [9], by definition offspring that inherit the same allele from both parents at this locus, assuming Mendelian inheritance including independent segregation, are identical by descent. The IAM locus also allows us to initialize the simulation without any positive spatial autocorrelation in genetic data. Therefore any spatial genetic structure that results over the course of the simulation is due to patterns of migration, drift, inbreeding, and local extinction.

### Landscape-scale accounting

LEA estimates the conservation value of alternative landscape configurations by including both demographic and genetic criteria. As described in detail previously [12], a credit represents the marginal contribution a change in landscape structure (i.e., taking or restoring of habitat) makes toward moving the ecological service closer to service levels observed in the recovery or pre-settlement landscape.

For the bank landscape, the number of LEA credits available in the bank at the time of the trade (t = 40) was estimated as Landscape Service Years (LSY) [12], which is a time-integrated estimate of the proportional change in ecological services relative to the sustainability goal due to marginal change in landscape structure. The number of abundance (N) credits is estimated as Landscape Service Years—Abundance (*LSY*_*C*_^*N*^):

$$LSY_{C}{}^{N}=\sum_{t=W}^{100}\left(\frac{m_{t}{}^{N}-j_{t}{}^{N}}{r_{t}^{N}}\right)$$ (1)

where *W* is the time the trade occurs, *r*_*t*_^*N*^ is the total abundance in breeding groups at year t provided by the recovery landscape, *m*_*t*_^*N*^ is the total abundance in breeding groups at year *t* provided by the bank landscape, and *j*_*t*_^*N*^ is the total abundance in breeding groups at year t provided by the baseline landscape.

The number of credits purchased to offset the local and regional loss of abundance due to a withdrawal, or the debit from the bank, can be calculated as Landscape Service Years—Abundance (*LSY*_*D*_^*N*^):

$$LSY_{D}{}^{N}=\sum_{t=W}^{100}\left(\frac{m_{t}{}^{N}-w_{t}{}^{N}}{r_{t}^{N}}\right)$$ (2)

where *w*_*t*_^*N*^ is the abundance at year *t* provided by the take landscape.

Calculating credits associated with changes in genetic variance is more complex. The management goal is to approximate population services provided by the distribution of habitat in which the organism evolved [34]. Greater genetic diversity within a breeding group or greater genetic divergence among breeding groups is not always better for sustainability [55]. The levels of genetic service provided by the pre-settlement landscape will be used to direct trading toward this goal. As estimates of genetic variance within and among breeding groups move closer to pre-settlement levels due to restoring habitat area or connectivity, the more credit is accrued in the bank. The credit representing a marginal change in genetic services associated with the mitigation landscape can be calculated as Landscape Service Years—Genetic Variance (*LSY*_*C*_^*G*^):

$$LSY_{C}{}^{G}=\sum_{t=W}^{100}\left(\frac{\left|p_{t}{}^{G}-j_{t}{}^{G}\right|}{p_{t}{}^{G}}\right)-\sum_{t=W}^{100}\left(\frac{\left|p_{t}{}^{G}-m_{t}{}^{G}\right|}{p_{t}{}^{G}}\right)$$ (3)

where *G* is the genetic variance component estimated (H_S_ or D_ST_), *p*_*t*_^*G*^ is the level of genetic variance at year *t* provided by the pre-settlement landscape, *j*_*t*_^*G*^ is the level of genetic variance at year t provided by the baseline landscape, and *m*_*t*_^*G*^ is the level of genetic variance at year *t* provided by the bank landscape. The first summation reports the extent of habitat fragmentation present in the landscape prior to addition of a bank. The second summation, to the right of the minus sign, reports the level of fragmentation after a bank is established. *LSY*_*C*_^*G*^ then equals the degree to which bank establishment reverses the effects of fragmentation.

The number of credits purchased to offset the take or departure of genetic variance away from pre-settlement-levels relative to bank-levels can be calculated as Landscape Service Years—Genetic Variance (*LSY*_*D*_^*G*^):

$$\begin{array}{l} E\left[LSY_{D}{}^{G}\right]=\sum_{t=W}^{100}\left(\frac{\left|p_{t}{}^{G}-w_{t}{}^{G}\right|}{p_{t}{}^{G}}\right)-\sum_{t=W}^{100}\left(\frac{\left|p_{t}{}^{G}-m_{t}{}^{G}\right|}{p_{t}{}^{G}}\right) \end{array}$$ (4)

where *w*_*t*_^*G*^ is the level of genetic variance at year t reflecting anticipated loss of habitat area or connectivity. *LSY*_*D*_^*G*^ equals the debit that results when the take moves the balance between recruitment and migration farther away from baseline levels.

### Managing stochasticity

In order to maximize convergence of model runs across stochastic simulations of landscape treatments, random permutation testing was used [56]. Random permutation was applied to distributions of N, Hs, and Dst at year 100 in replicate runs of the landscape treatments that included the most landscape change (i.e., usually the take treatment). Starting at 100, Monte Carlo iterations were then increased until no significant difference (p>0.01) between replicate runs of a landscape treatment were found. This resulted in running 100 iterations for the pre-settlement, 800 for the recovery, and 1,500 iterations for all remaining landscape treatments.

Bootstrapping was applied to estimate 10,000 credit values by resampling the distribution of 100, 800, or 1,500 possible outcomes at random with replacement for each landscape treatment to characterize and compare the distributions of credit values. Random draws were used for each year to pick N, Hs, and Dst from each landscape treatment to be applied to eqs 1–4. Results from these distributions were summarized based on the number of credits remaining after a trade (i.e., credits—debits). Number of LSY remaining is a time-integrated estimate of the net gain or loss of ecological services due to the change in landscape structure. Application of random permutation testing to the distribution of remaining credits (n = 10,000) found significant differences (p<0.01) among almost all take landscape treatments, which is not surprising due to sample size tested. Rather than present statistical summaries of all pairwise comparisons, I chose to plot the distributions of bootstrapped values.

### Value of information

Decision analysis [40, 41] was applied to characterize how uncertainty regarding dispersal behaviors affects decision making. Trades were compared based on the median number of LSY credits remaining for each of the three ecological services after a trade. The most simple way to reduce the dimensionality of results is to calculate the Expected Value (EV) of a trade given uncertainty in dispersal behaviors captured by the five models selected by POM as:

$$EV\left[T|M\right]=\sum_{M=1}^{5}P[M]*LSY_{M,T}$$ (5)

Where, T is the trade considered, M is the index for the five uncertain models, P[M] is the prior probability that a parameterization is true, LSYM,T is the number of LSY credits remaining (debits-credits). Each model was assumed to have an equal probability of being true, P[M] = 0.2, because the POM technique applied a threshold criteria to identify the five best models [39]. The trade with the greatest expected value is referred to as the preferred trade (EV[T_p_|M]).

Next decision analysis estimates the expected credits remaining assuming that the best trade is made given the expectations under each model. Thus, the expected value of knowing the true dispersal behaviors (i.e., the true model) given the possible trades available:

$$EV\left[M|T\right]=\sum_{M=1}^{5}P[M]*\text{max}\left(LSY_{M,T}\right)$$ (6)

Where, max(LSYM,T) is the largest number of LSYs remaining from the trades compared for each model. If EV[M|T] does not equal EV[T|M] for all models, then at least one of the five models indicates that a greater conservation benefit can be achieved by making a different trade than suggested when considering all models to be equally likely.

The expected value of learning the true dispersal behaviors can now be estimated as the expected value of perfect information (*EVPI* = *EV*[*M*|*T*] − *EV*[*T*_*p*_|*M*]). If EVPI is positive then reducing uncertainty, through further data collection, may lead to a different trade that provides greater conservation benefits.

To value the new information acquired by POM relative to the old information provided by model 6(DSS), I calculated EVPI relative to model 6(DSS) (EVPI^DSS^) for the trade providing the highest EVPI for abundance credits [42]. EVPI^DSS^ was simply estimated as *EV*[*M*|*T*] − *EV*[*T*_*p*_|*DSS*], where EV[M|T] is the expected value of knowing the true dispersal model included in the POM set, as estimated above.

### Pricing credits

Prices for credits in the U.S. conservation banking market are set by firms usually operating for-profit businesses. For this analysis we assume that bankers cover operating costs (i.e., purchase of land, habitat restoration costs, habitat management costs, and the cost to fund a non-wasting endowment) plus 8% profit (S1 Table).

Given the strong impact that habitat fragmentation, independent of habitat amount, has on genetic diversity, and strong impact of habitat area, independent of habitat fragmentation, has on abundance [9], I assumed that network externalities would be estimated using credits for Hs and Dst alone. There is currently no regulatory basis for trading credits for genetic diversity within a population [13]. Here, I apply genetic criteria as only a cap to examine the possible contribution recognizing network externalities can make to mitigation markets. Therefore, trades mustn’t produce an allocation of habitat that drives the spatial apportionment of genetic variation farther away from pre-settlement than is observed under a baseline habitat allocation leading to a negative balance of credits remaining for Hs or Dst [12]. Presentation of genetic credits remaining is included to contrast the genetic credits relative to those observed for abundance. I assume that bankers seek to recoup costs based on credits for abundance alone, as these credits represent avoidance of take. The price of credits was estimated simply as the revenue target of the banker divided by available credits for abundance (LSY N, eq 1) expected by creating a bank.

## Results and Discussion

### Price of credits

**Fig 2:**
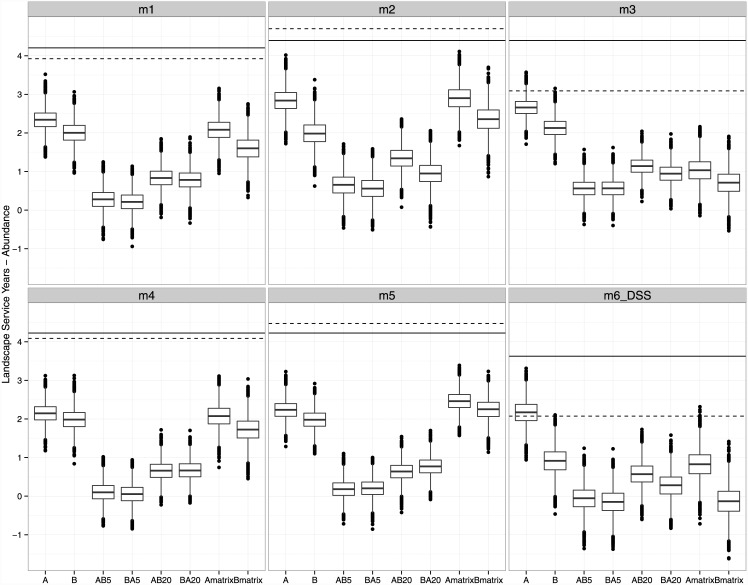
Landscape Service Years for abundance (N). Solid horizontal line represents the number of credits available in the bank, prior to a trade, assuming a static matrix and the dashed horizontal line is the number of credits available under a declining matrix. Boxplots of bootstrapped credit values remaining after each trade, with bars represent median values, the box represents the upper and lower 25^th^ quartile, whiskers represent max/min values excluding outliers, and dots represent outliers, or values 1.5 x 25^th^ quartile.

The total revenue expected by the banker to offset costs to restore and maintain 25 territories plus 8% profit would be $22,342,733 (S1 Table). Credits available in the bank (eq 1) varied across the five dispersal models selected by inverse parameterization and whether a static or declining matrix was simulated (Fig 2). These values ranged from 3.09 to 4.70 LSYs, with the minimum and maximum both occurring under a declining matrix. To estimate the price of credits for abundance, I assumed that a banker would minimize risk by pricing credits based on the maximum possible price recognizing that matrix habitat may decline over time. Therefore, the price of a LSY for abundance would be $7,231,200/LSY N (i.e., based on the fewest credits observed with a declining matrix). This price represents the in-kind replacement costs for abundance estimated at a landscape scale.

### Bank value

Abundance credits available in the bank (Fig 2) were more often greater when the matrix habitat was static, indicating that dispersal is contributing more to population growth with a static matrix. In contrast, genetic credits available in the bank were more often greater under a declining matrix, indicating that the bank is contributing more to reducing rates of drift and inbreeding averaged across the landscape when dispersal habitat is lost over time.

### Trading, transient dynamics and abundance credits

For abundance, there was a net decrease in the number of credits in the bank due to the loss of habitat in areas A and B, regardless of whether takes occurred individually or in series, or whether matrix habitat was static or decreasing (Fig 2). Though both take A and B affected the same number of territories, more credits remain after take A than B (Table 2). The difference in credits remaining comparing the loss of area A or B, especially for model 2, model 3, and model 6(DSS), indicates that landscape processes (i.e., not just patch area) are important determinants of abundance. While the difference between A and B is only 0.41 LSY N credits remaining (Table 2) that translates to a financial difference of $3,017,678. The differences in credit values remaining for take A versus B could reflect differences in the number of helpers in the take areas, or due to changes in habitat occupancy in nearby territories not the subject of take but affected by changes in dispersal patterns [13].

**Table 2:** Results of Decision Analysis to determine the Expected Value of Perfect Information regarding how land use affects dispersal behaviors. Values are estimated as LSYs remaining and as financial values assuming the price of a credit for abundance (LSY N) is $7,231,200. Financial value of credits for genetic diversity has not yet been estimated by these markets.

		N	Hs	Dst
**A vs. B**	EV[A|M]	2.46	1.36	3.54
	EV[B|M]	2.02	0.621	1.64
	EVPI	0	0	0
	EVPI—$	$0		
**Amatrix vs. Bmatrix**	EV[Amatrix|M]	2.21	1.49	4.46
	EV[Bmatrix|M]	1.83	0.816	3.01
	EVPI	0	0	0
	EVPI—$	$0		
**Amatrix vs. A**	EV[A|M]	2.46	1.36	3.54
	EV[Amatrix|M]	2.21	1.49	4.46
	EVPI	0.0646	0.222	0.391
	EVPI—$	$467,495		
**Bmatrix vs. B**	EV[B|M]	2.02	0.621	1.64
	EV[Bmatrix|M]	1.83	0.816	3.01
	EVPI	0.137	0.117	0.00462
	EVPI—$	$987,086		
**AB5 vs. BA5**	EV[AB5|M]	0.356	0.895	2.71
	EV[BA5|M]	0.336	0.834	2.53
	EVPI	0.00615	0	0
	EVPI—$	$44,485		
**AB20 vs. BA20**	EV[AB20|M]	0.936	1.10	3.14
	EV[BA20|M]	0.835	0.795	2.29
	EVPI	0.0243	0	0
	EVPI—$	$175,988		
**AB5 vs. AB20**	EV[AB5|M]	0.356	0.895	2.71
	EV[AB20|M]	0.936	1.10	3.14
	EVPI	0	0	0
	EVPI—$	$0		
**BA5 vs. BA20**	EV[BA5|M]	0.336	0.834	2.53
	EV[BA20|M]	0.835	0.795	2.29
	EVPI	0	0.0147	0
	EVPI—$	$0		

When considering two takes occurring in series separated by five years, assuming a static matrix (i.e., AB5 and BA5), the median credits remaining for abundance were close to zero for all models selected by POM, and less than zero for model 6(DSS). Allowing more time between takes (i.e., AB20 and BA20) increased the number of abundance credits remaining for all models. Therefore, landscape transient dynamics affect abundance credits. The influence of transient dynamics on abundance indicates why basing trades on geometric indices indicative of the relationship between landscape and biodiversity patterns at one moment in time will be unable to predict the influence of landscape change in the future (i.e., such analysis ignores the role of landscape history). However, bootstrapped distributions of remaining credit values were often similar regardless of whether take A proceeded B, or vice versa, though allowing more time between trades led to greater differences between the AB and BA treatments. Whether the remaining credits increased, decreased, or stayed about the same due to declining matrix habitat varied by dispersal model.

### Trading, transient dynamics and genetic credits

**Fig 3:**
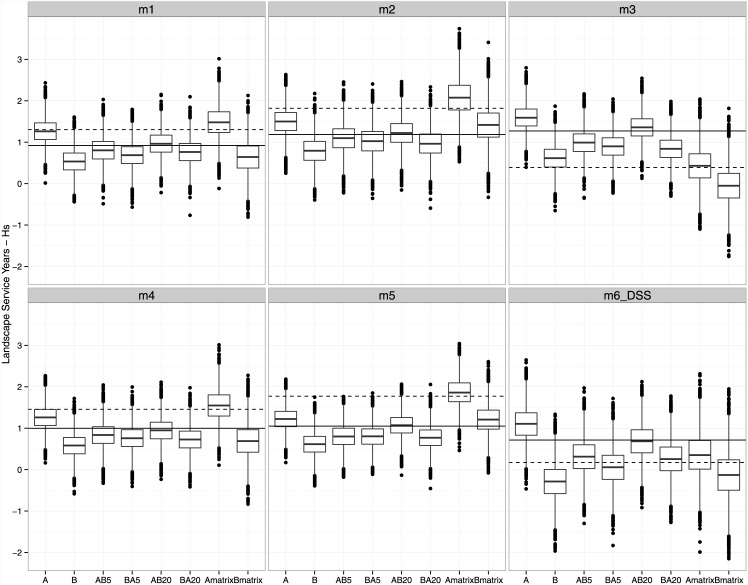
Landscape Service Years for average expected heterozygosity within breeding groups (Hs). Solid horizontal line represents the number of credits available in the bank, prior to a trade, assuming a static matrix and the dashed horizontal line is the number of credits available under a declining matrix. Boxplots of bootstrapped credit values remaining after each trade, with bars represent median values, the box represents the upper and lower 25^th^ quartile, whiskers represent max/min values excluding outliers, and dots represent outliers, or values 1.5 x 25^th^ quartile.

**Fig 4:**
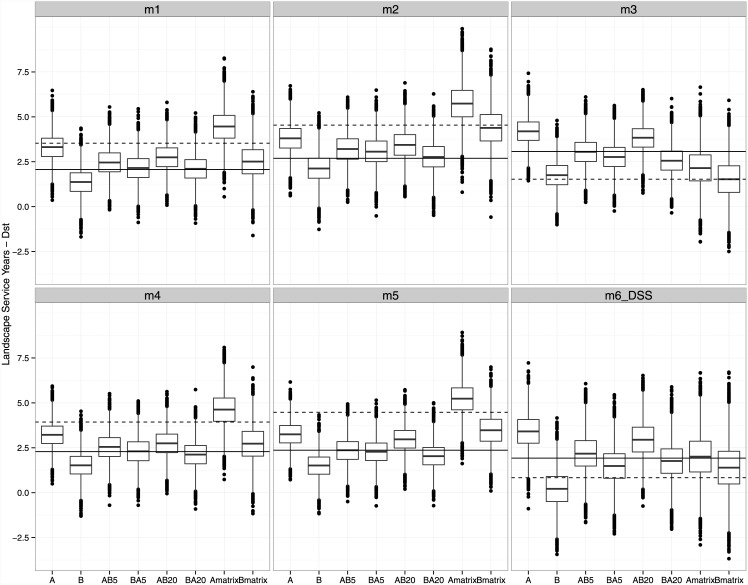
Landscape Service Years for average genetic divergence among breeding groups (Dst). Solid horizontal line represents the number of credits available in the bank, prior to a trade, assuming a static matrix and the dashed horizontal line is the number of credits available under a declining matrix. Boxplots of bootstrapped credit values remaining after each trade, with bars represent median values, the box represents the upper and lower 25^th^ quartile, whiskers represent max/min values excluding outliers, and dots represent outliers, or values 1.5 x 25^th^ quartile.

Whether a take resulted in a net decrease or increase in credits for genetic diversity, relative to the number of credits available in the bank (i.e., a positive or negative network externality), often varied by take and dispersal parameterization (Figs 3 and 4). One exception was the expected change in network externality caused by take A and B individually and assuming a static matrix. In this case, all models predict a net gain in genetic credits under take A but a net decrease under take B. This was also the case assuming a declining matrix except for model 6 (DSS) for Dst credits, in which a positive network externality for both A and B based on median values was observed.

When takes A and B occurred in series, a median net increase in credits for Dst was often observed when A was taken before B, and no change or a slight decrease in credits was often observed when B preceded A. In contrast, credits for Hs often indicated a net decline in median credits relative to the number of credits available in the bank regardless of whether A preceded B or vice versa. It was surprising to see that the time interval between takes differing by as a little as five years or as much as 20 years did not strongly affect the magnitude of network externality. The order of trades had a more important impact on network externalities, determining whether network externalities are positive or negative. Therefore, the order of takes affects conservation value of connectivity. These results agree with a recent study of tiger salamanders in which wetland area and connectivity had a bigger influence on genetic divergence among breeding populations than wetland age or hydroperiod [57].

The presence of a declining habitat matrix affected genetic credit values more under some dispersal models than others. For model 3 and trade B under a declining matrix (i.e., Bmatrix), an increase in fragmentation effect was observed, as estimated by median credit values for Hs < 0, and yet a positive balance for abundance credits remained. Therefore, if median values for genetic credits were used to assess if the cap on network externalities was violated, then trade B would not be a viable trade if the habitat matrix were expected to decline. Therefore, the loss of unregulated dispersal habitat may prevent mitigation banking from protecting conservation values.

Results for genetic credits indicated that network externalities may be positive or negative depending on the order in which the trades occur. Large dimensionality of spatial problems is often the barrier to applying spatially- and temporally-explicit techniques [29, 31], but the integration of POM and LEA here provides one solution for mitigation banking. Granted, had I attempted to distribute subsidies over many landowners at one point in time [29], the curse of dimensionality would remain but the temporal differences would not matter if all landowners could supply conservation values at the same time. Mitigation banking markets often entail one, or a few sellers, and buyers pursuing development at specific times, reducing the dimensionality.

### Value of learning

Decision analysis indicated that when comparing treatments assuming static matrix habitat, that the EVPI was often zero for abundance credits (Table 2). The exception was when determining the best order of trades when separated by five or 20 years (AB5 vs BA5 and AB20 vs BA20). Therefore, dispersal models disagree regarding which order of trades would lead to the greatest number of remaining credits for both intervals. For example, the value of knowing, before a trade occurs, how RCW dispersal behaviors will interact with a new landscape pattern generated by a trade is $175,988 when comparing trades AB20 vs BA20. EVPI represents the amount of money that could be saved if further research indicated that the dispersal model leading to the greatest LSYs remaining under each trade is indeed the true model. Therefore, it provides a reasonable approximation for the maximum amount we should spend on further data collection and analysis.

The value of learning the true dispersal model (i.e., EVPI), based on abundance credits, was greatest when contrasting take A or B in static versus a declining matrix. Given the price of credits, this leads to the estimated value of learning the correct dispersal behaviors to be $467k to $987k, with the greatest value observed when contrasting take B in static versus declining matrix. Thus, differences in landscape change (i.e., take A vs. B) affect EVPI. In other words, the magnitude with which dispersal models disagree regarding which is the best trade will vary by the landscape change being compared. It is important to note that the an EVPI of $877k (Bmatrix vs B), does not mean that if we spent that sum on collecting more data that we are guaranteed to save $877k by making smarter trades thanks to a more certain model. EVPI represents the amount of money that could be saved if further research indicated that the dispersal model leading to the greatest LSYs remaining under each trade is indeed the true model. From this perspective it is more compelling that the comparison of most trades indicated an EVPI of zero, indicating the usefulness of POM and contradicting the early criticisms of IB-SEPMs [37, 58].

For genetic credits, EVPI was greatest when comparing take A assuming a static versus a declining matrix. In fact, results indicated that EVPI, measured in LSYs, was greater for genetic credits than abundance credits. Therefore, there is greater disagreement among dispersal models regarding the impact a declining matrix would have on the retention of genetic diversity within and among breeding groups for take A.

Interestingly, EVPI for Hs credits were greater than zero when determining whether it would be better to separate take B followed by A by five or 20 years (i.e., BA5 vs BA20). Therefore, while it is clearly best for abundance to allow for more time between trades, we are uncertain if allowing more time would lead to a greater retention of average expected heterozygosity within breeding groups.

If the revenues from EV[T|M] are sufficient and the probability of no net loss of abundance and genetic diversity are high (e.g., AB5 or BA5), bankers might decide to forego data collection, even if EVPI>0, and implement trades. After all, bankers will want to sell as many credits as they can, as fast as they can. Alternatively, if EVPI were high and the probability of no net loss of abundance or genetic diversity were low (i.e., Bmatrix), the present analysis provides economic and ecological justification to forego a trade and collect more data.

The value of applying 13-years of monitoring data within an IB-SEPM using POM relative to making decisions using past knowledge (DSS) was assessed by the estimate for EVPI^DSS^. The greatest EVPI for abundance was observed for Bmatrix vs B. Therefore, I calculated EVPI^DSS^ based on the comparison of these trades. EVPI^DSS^ was 0.989 LSYs N (*EV*[*M*|*T*](2.16) − *EV*[*B*|*DSS*](1.17)), which is seven times greater than EVPI estimated using current information (0.137 LSYs N) for trades Bmatrix vs B. Using the banker’s current estimate of prices for abundance credits that would be a potential savings of $7.15 million. This value represents the cost of making a decision while ignoring uncertainty and lacking perfect knowledge [51]. Therefore, reducing model uncertainty by applying 13-years of monitoring data reduced EVPI by ~$6.2 million (i.e., $7.15 million–$0.877 million). This value represents the decrease in potential lost revenues due to dispersal uncertainty, and provides an upper bound for valuing the 13-yr monitoring program and the application of LEA and POM for trade B given uncertainty in the loss of dispersal habitat. The majority of data used in Bruggeman et al. [39] were collected by Federal agencies wishing to have adequate data on hand to address potential Section 7 consultations under ESA, as recommended by the species recovery plan [21].

## Conclusion

These results indicate the importance of spatial-, temporal-dynamics for conservation banking markets. To the best of my knowledge this represents the first comparison of changes in abundance and genetic diversity assuming dynamic breeding and dispersal habitat, and including spatial-, temporal- feedback mechanisms. It would be best to revise the conservation banking guidance to, at a minimum, encourage the use of spatially- and temporally-explicit techniques that estimate changes in population genetic metrics at a landscape-scale.

While it has long been recognized that establishment of a bank can lead to habitat defragmentation, we have shown here that habitat trades themselves (i.e., a take) can lead to habitat defragmentation estimated using population genetic theory. This poses policy challenges on four fronts. First, traditional interpretations of take and jeopardy do not include changes in network externalities [12]. Second, and consequently, there are no mechanisms within a mitigation market to benefit from the potentially large producer surplus in network externalities generated by habitat trading. Third, landscape scale management requires a holistic approach recognizing the role of multiple land uses, not just foraging and breeding habitat, to achieve no net loss of abundance and genetic diversity. Fourth, simple trading currencies based on habitat geometry, even if they incorporate a species’ dispersal distance, will likely miss important information because the order of trades affects whether network externalities will be positive or negative.

No requirement for validation is included when determining if science is “available” under the ESA, so tradable credit systems could be developed using methods that have not been shown to be predictive of habitat occupancy or genetic diversity in dynamic landscapes. If we are willing to exchange habitat for such populations then we should also be willing to test our assumptions about how land use change affects population viability. If available data are excluded when evaluating trades due to administrative constraints, then an understanding of the potential conservation cost of using simple metrics should at least be expressed.

Mitigation banking trades uncertain benefits for certain losses [59]. Therefore, unlike warnings regarding decisions to preserve habitat [60], delaying action (i.e., habitat loss) in order to learn, may lead to improved mitigation outcomes and increased cost-effectiveness of management. Grantham et al. [60] argued that making conservation decisions based on habitat maps and not spending more than a year or two collecting monitoring data maybe the best conservation strategy. However, it should be noted that Grantham et al. [60] ignored spatial-, temporal- feedback mechanisms that determine species diversity. Therefore, the contribution monitoring data make to learning about the biological processes that underlie biodiversity in dynamic landscapes was not assessed. Therefore, an adequate modeling framework is needed to spur learning and to assign value to monitoring data.

Others have contrasted the ability of IB-SEPMs to inform landscape management decisions with more simple landscape models, some have found important differences [61] while others have concluded that simple models perform sufficiently well for decision making [62]. The different conclusions may result from the experimental approach taken. Jepsen et al. [61] contrasted the ranking of alternative habitat patch configurations generated by two IB-SEPMs (i.e., assuming static and dynamic landscape conditions) and more simple models including a metapopulation model and an individual movement model (i.e., simulation of movement but not reproduction). Only the dynamic IB-SEPM ranked habitat patch configurations differently, while static IB-SEPM, metapopulation model, and individual movement model were largely in agreement. Minor and Urban [62] contrasted the importance of patches within a landscape using a static IB-SEPM and graph theory. Graph theory distils a map into a set of nodes that represent the placement of habitat patches and edges that describe connectivity of nodes. Minor and Urban [62] found strong correlation among the ranking of patches from both the static IB-SEPM and Graph Theory. The difference between Jepsen et al. [61] and Minor and Urban [62] may have resulted from the absence of landscape dynamics in the Minor and Urban [62] study.

Granted, RCW management has benefited from the availability of an IB-SEPM constructed with 15-years of monitoring data [44], which in market terms subsidized the transaction costs of this hypothetical market. What if such a model were unavailable? Traditionally, to account for uncertainty regarding the ecological equivalence of conservation bank and take areas, mitigation ratios are applied such that a bank must be “x” times larger than the take [15]. Moilanen et al. [59] found that very large mitigation ratios—on the order of 10:1 to 100:1—maybe needed to prevent a net loss of biodiversity given uncertainty in conservation benefits at a bank. Such large ratios would prevent the creation of a market for species such as RCWs in which restoration and management costs are high. The present study demonstrates that using an IB-SEPM and POM to manage uncertainty may allow ratios <1:1 (i.e, ratio for trade AB = 25:28 territories) if trades can reduce habitat fragmentation effects. Therefore, while detailed natural history information available for RCWs will often not be available for other species, it may be financially beneficial to begin constructing even simple IB-SEPMs and begin testing model validity with monitoring data as the landscape changes over time due to trading. It would also provide an opportunity to understand the potential demographic and genetic implications of using simple metrics.

The integration of POM and LEA outlined here would be useful in passive adaptive management. Adaptive management is a method of incorporating the role of uncertainty in decision-making by treating decisions as hypotheses regarding the system’s response to management actions [63]. Passive adaptive management would use monitoring data collected over time, as trading progresses, to test assumptions of the IB-SEPM using POM and the new set of parameters would be used to evaluate future trades.

When working to ensure trades do not decrease population viability at a landscape scale, our ability to monitor and detect changes in rates of migration, drift, inbreeding, and local extinction will be limited, a problem referred to as partial observability [64]. Recall that EVPI, estimated as LSYs, was often greater for genetic credits than abundance credits indicating that genetic diversity is providing an important information service. In other words, the values for EVPI suggest that the five models led to more divergent estimates of genetic diversity than abundance. Bruggeman et al. [39] found genetic diversity had the lowest information content for estimating dispersal, but their study was restricted to genetic data from a small spatial extent. Therefore, a genetic monitoring program implemented at a broad spatial scale may provide adequate information content. It may be more cost-effective to estimate changes in rates of recruitment and migration across the landscape by confronting an IB-SEPM with population genetic data than mark-recapture and/or radio telemetry studies—though a mix of monitoring data would be best [39].

Walker et al. [4] argued that institutional limitations (e.g., limited data collection and model development) and the strong economic drivers to use simple trading metrics will prevent markets for biodiversity from obtaining conservation goals. History has proven Walker et al. [4] correct, but their position is based on political and ecological theory, and they failed to include any economic data or techniques. Here, I have integrated landscape ecology, natural resource economics and decision analysis to highlight when a lack of information prevents cost-effective decision making. Whether conservation institutions could effectively develop and apply spatially-, temporally-explicit models to manage complexity and evaluate trades remains to be seen, but here I provide evidence that such models can be used to align financial and conservation benefits of tradable credits systems. The LEA and POM tools developed here would support President Obama’s recent call [65] for equivalent standards for landscape-scale mitigation across Federal agencies that lead to resilient ecological and economic outcomes.

## Supporting Information

S1 Appendix Description of the individual-based, spatially-explicit population model for the Red-cockaded Woodpecker (DOC)

S1 Table Estimated costs and profits for the hypothetical RCW conservation bank with 25 territories (DOC)
