# Supplementary material for: The Value of Learning about Natural History in Biodiversity Markets
Source: PLoS One. 2015 Dec 16;10(12):e0144047. doi: 10.1371/journal.pone.0144047 (PMC4684417; doi:10.1371/journal.pone.0144047)
Supplement: S1 Appendix — (DOC) [file pone.0144047.s001.doc]

# S1 Appendix

# Description of the Individual-based, Spatially-Explicit Population Model for the red-cockaded woodpecker

Details of the RCW IB-SEPM are described below following the Overview, Design concepts, and Details (ODD) protocol, which is intended to provide a standard approach for describing agent-based models across studies to increase transparency (Grimm et al. 2010).

Table of Contents

[1. Purpose 2](#__RefHeading___Toc407290960)

[2. Entities, state variables, and scales 2](#__RefHeading___Toc407290961)

[3. Process overview and scheduling 4](#__RefHeading___Toc407290962)

[4. Design concepts 4](#__RefHeading___Toc407290963)

[5. Initialization / Constraining factors 7](#__RefHeading___Toc407290964)

[6. Input data 8](#__RefHeading___Toc407290965)

[7. Submodels 9](#__RefHeading___Toc407290966)

[8. References 13](#__RefHeading___Toc407290967)

# 1. Purpose

The purpose of the Red-cockaded Woodpecker (RCW) IB-SEPM, originally described in Letcher et al. (1998) and updated here, is to model a complex social system that includes spatially restricted dispersal. The purpose of the model is to estimate how landscape change may affect population viability and use Landscape Equivalency Analysis (LEA) to estimate the conservation value of alternative landscape compositions (Bruggeman et al. 2005).

# 2. Entities, state variables, and scales

*Breeding groups*. The RCW IB-SEPM simulates the cooperative breeding system of RCWs. Breeding groups consist of male and female breeders, fledglings, and, helpers who are usually male and full or half-sibs to the fledglings (Figure A; Walters et al. 1988). Male helpers play a critical role in population dynamics by participating in the defense of the territories, feeding of nestlings, and inheriting their natal territory upon the death of the male breeder. Male helpers will preferentially inherit their natal territory upon the death of the breeding male, out-competing floaters and helpers in adjacent territories [described further below]. In contrast, we are more uncertain of the role female helpers play, but review of bird banding data indicate they do not inherit their mother‘s role as a breeder in their natal territory – so this was excluded from the model. Floaters of both sexes are also present in the region, which move continuously seeking a breeding vacancy in a territory (Walters et al., 1988).

Female

breeder

Male

Fledgling(s)

Helper(s)

Breeder

Competition

Inbreeding Avoid.

Female

Fledgling(s)

**Floater**

Male

breeder

**Figure A**. Breeding group structure and demographic transitions for the Red-cockaded Woodpecker. Black boxes denote breeding territories and the grey box denotes the matrix crossed during floating behaviors. Over 90% of time when a helper inherits his father’s territory, his mother disperses to avoid inbreeding (Daniels and Walters, 2000).

*Agents/individuals.* The basic entity modeled is a bird. Each bird is characterized by its sex, age, status (i.e., fledgling, floater, helper, or breeder), current location, natal territory location, and alleles present at four genetic loci. If it is a floater, its dispersal direction, dispersal path, and number of steps available to be taken within a season are tracked. If it is a breeder, its breeding territory location and number of fledglings produced are tracked. If it is a potential competitor for a breeding vacancy (i.e., a helper or floater), the bird’s perceptual distance is also tracked.

*Landscape.* The hypothetical landscape covered 547,600 ha, and each cell is a 100 m x 100 m or 1 hectare. The simulations included land cover classified as high quality habitat (mimicking Longleaf pine savannah that serves as breeding and foraging habitat [Walters et al. 2002]), matrix habitat mostly used for movement (i.e., mixed pine or hardwood forests, referred to here as matrix), and non-forested areas, which may serve as barriers during movement (Bruggeman et al. 2009).

*Time.* The simulation experiment was run for 100 years. Four seasonal time steps were simulated within each year, starting with Spring. The seasons corresponded to Spring: April, May, June; Summer: July, August, September; Fall: October, November, December; and Winter: January, February, March.

# 3. Process overview and scheduling

Pseudo-code describing the model scheduling is provided below and is largely based on Letcher et al. (1998).

| Read data files (parameter values, landscape, territory locations by year, shapefiles describe habitat loss and restoration)  For parameterization p  For monte carlo m  For land L  For year t=1 to 100  If t=0, Initialize territories with RCWs  Update available territories  If L=mitigation  Add Bank territories for natural colonization  For season s  If s=Spring  Reproduction  Add Immigrant, every 4 years  Age birds  Estimate genetic parameters [Nei]  Population census  End if Spring  Mortality  If s>Spring, Natal dispersal  Competition  Dispersal  End season  End t [year]  End L  End m  Collect landscape-scale data for LEA  End p |
| --- |

# 4. Design concepts

The basic principles underlying this model’s design are the associations between demographic and genetic components of population structure in a dynamic landscape. The design will allow us to understand how demographic and genetic properties relate in dynamic landscapes. Demographic stochasticity is included in the mortality, reproduction, competition, and dispersal submodels described below. The model includes a hypothetical genetic loci and population genetic characteristics are summarized following the method of Nei (1973) coded directly within the simulation, discussed further below under Submodels.

Landscape Equivalency Analysis (LEA) is an extension of resource-based compensation applied to a landscape-scale (Bruggeman et al 2005). Resource-based compensation determines the amount of restoration required to equate an individual’s well-being before loss of an ecological resource with their well-being after that loss (Jones and Pease 1997). A “service-to-service” approach is used wherein adequate compensation is made to the public if the habitats restored provide equivalent types and levels of ecological services as the habitats lost. Ecological functions can be treated as goods and services when a direct or indirect benefit to humans can be demonstrated (deGroot et al 2002). In this analysis we assume that the ecological services that provide these benefits are abundance and genetic variance (Loomis and White 1996; Bruggeman et al 2005). LEA estimates the equivalency of habitat patches traded in a fragmented landscape based on changes in three ecological services, 1) abundance and genetic variance 2) within and 3) among local populations, measured at the landscape-scale.

The spatial apportionment of neutral genetic variance is included to ensure that trades do not move the **balance** between genetic drift, local extinction, and migration farther away from that observed under a “baseline” landscape. Neutral genetic variance is the variety of alleles present in the population that do not contribute to adaptive traits and whose fate is determined by genetic drift, gene flow, inbreeding, and local extinction (Manel et al 2003). Population genetic studies have shown that spatially subdivided populations benefit from migration rates that are high enough to episodically contribute to recruitment, thereby lowering rates of loss of genetic diversity and rates of inbreeding accrual, but low enough to maintain genetic differences among breeding groups to provide opportunities for adaptive evolution (Mills and Allendorf 1996; Wang 2004). However, the appropriate amount of migration required to offset genetic drift and inbreeding will vary based on an organism’s natural history and the degree of habitat loss and fragmentation at the landscape level (Lacy and Lindenmayer 1995).

Spatially subdivided populations in intact landscapes have demonstrated an ability to maintain genetic variance both within and among breeding groups while preventing inbreeding depression (reviewed in Bruggeman et al 2005). Therefore, LEA incorporates a “spatially-explicit” planning objective as the allocation of habitat yielding the spatial apportionment of neutral genetic variance observed prior to habitat loss and fragmentation (i.e., a baseline landscape) (Meffe 1996; Bruggeman et al 2005). The baseline landscape represents the spatial distribution of habitat in which rates of recruitment and migration achieve this balance. The conservation value of a LEA credit increases as habitat trades are able to move the balance between rates of recruitment and migration closer to levels observed prior to habitat loss and fragmentation, or a “baseline” apportionment of habitat. This reduces the probability that changes in recruitment and migration would lead to the expression of deleterious traits that result from mating between close relatives (inbreeding depression). Similarly, this reduces the probability that changes in migration would disrupt locally adapted gene complexes due to mating between individuals from different regions (outbreeding depression). In this way, LEA focuses on protecting the environmental context that permitted adaptive evolution, rather than attempting to identify all adaptive components of genetic variance (Moritz 2002).

A LEA credit represents the marginal contribution a change in landscape structure (i.e., taking or restoring of habitat) makes toward moving the ecological service closer to service levels observed in the recovery or pre-settlement landscape. For the bank landscape, the number of LEA credits available in the bank at the time of the trade (t=40) was estimated as Landscape Service Years (LSY; Bruggeman et al 2005), which is a time-integrated estimate of the proportional change in ecological services relative to the sustainability goal due to marginal change in landscape structure. The number of abundance credits is estimated as Landscape Service Years - Abundance (*LSYCN*):

$$LSY_{C}^{N}=\sum_{t=W}^{100} \left(\frac{m_{t}^{N}-j_{t}^{N}}{r_{t}^{N}}\right)$$

where *W* is the time the trade occurs, *rtN* is the total abundance in breeding groups at year t provided by the recovery landscape, *mtN* is the total abundance in breeding groups at year *t* provided by the bank landscape, and *jtN* is the total abundance in breeding groups at year t provided by the baseline landscape.

The number of credits purchased to offset the local and regional loss of abundance due to a withdrawal, or the debit from the bank, can be calculated as Landscape Service Years - Abundance (*LSYDN*):

$$LSY_{D}^{N}=\sum_{t=W}^{100} \left(\frac{m_{t}^{N}-w_{t}^{N}}{r_{t}^{N}}\right)$$

where *wtN* is the abundance at year *t* provided by the take landscape.

Calculating credits associated with changes in genetic variance is more complex. The management goal is to approximate population services provided by the distribution of habitat in which the organism evolved (Meffe 1996). Greater genetic diversity within a breeding group or greater genetic divergence among breeding groups is not always better for sustainability (Bouzat 2001). The levels of genetic service provided by the pre-settlement landscape will be used to direct trading toward this goal. As estimates of genetic variance within and among breeding groups move closer to pre-settlement levels due to restoring habitat area or connectivity, the more credit is accrued in the bank. The credit representing a marginal change in genetic services associated with the mitigation landscape can be calculated as Landscape Service Years – Genetic Variance (*LSYCG*):

$$LSY_{C}^{G}=\sum_{t=W}^{100} \left(\frac{\left|p_{t}^{G}-j_{t}^{G}\right|}{p_{t}^{G}}\right)-\sum_{t=W}^{100} \left(\frac{\left|p_{t}^{G}-m_{t}^{G}\right|}{p_{t}^{G}}\right)$$

where *G* is the genetic variance component estimated (HS or DST), *ptG* is the level of genetic variance at year *t* provided by the pre-settlement landscape, *jtG* is the level of genetic variance at year t provided by the baseline landscape, and *mtG* is the level of genetic variance at year *t* provided by the bank landscape. The first summation reports the extent of habitat fragmentation present in the landscape prior to addition of a bank. The second summation, to the right of the minus sign, reports the level of fragmentation after a bank is established. *LSYCG* then equals the degree to which bank establishment reverses the effects of fragmentation.

The number of credits purchased to offset the take or departure of genetic variance away from pre-settlement-levels relative to bank-levels can be calculated as Landscape Service Years – Genetic Variance (*LSYDG*):

$$\begin{matrix} E\left[LSY_{D}^{G}\right]=\sum_{t=W}^{100} \left(\frac{\left|p_{t}^{G}-w_{t}^{G}\right|}{p_{t}^{G}}\right)-\sum_{t=W}^{100} \left(\frac{\left|p_{t}^{G}-m_{t}^{G}\right|}{p_{t}^{G}}\right) \\ \end{matrix}$$

where *wtG* is the level of genetic variance at year t reflecting anticipated loss of habitat area or connectivity. *LSYDG* equals the debit that results when the take moves the balance between recruitment and migration farther away from baseline levels.

# 5. Initialization / Constraining factors

At the start of each simulation, 80% of clusters were selected at random and given a breeding pair. Of these, 50% were randomly chosen to have one male helper. Age was also randomly assigned at the start of each Monte Carlo simulation by choosing from a normal distribution with a mean of 4 and standard deviation of 1. All birds were assigned alleles at one hypothetical genetic locus. It was assumed that every founding individual is heterozygous and contains two unique alleles (i.e., total alleles = 2 x number of breeders in the founding population, an Infinite Alleles Model (IAM) of genetic variation).

To allocate RCW territories across each landscape, we first completed a review home range studies. We define home range as the area used for breeding and foraging, but all of the foraging habitat is not necessarily defended against other RCW groups. Twenty-one studies were identified. The majority of home range studies used the Minimum Convex Polygon (MCP) method to calculate home range, but many have argued that the Fixed Kernel method is a more accurate estimate of RCW habitat (Convery 2002; Franzreb 2006; Wood 2008). The Fixed Kernel method uses a probabilistic approach to determine home range size based on the density of RCW observations made while following birds during foraging. Home range tends to decrease during nesting season when adults forage closer to the nest. We felt the best estimates for home range size were those obtained outside of the nesting season and using a Fixed Kernel method (i.e., Convery 2002; Walters et al. 2002; Franzreb 2006). The mean home range size across these studies was 73 ha (min = 56; max = 128). Two of the studies reported a standard deviation of 5 hectares.

Therefore, we created a toolbox that randomly assigns RCW territories to the pre-settlement landscape by choosing a territory center at least 400 m from the nearest neighboring cluster. The distance criterion is based on a USFWS RCW management standard (USFWS 2003). Then the habitat area for each territory was randomly selected from a normal distribution (mean = 73 and standard deviation = 5). To accept a randomly placed cluster, there had to be enough RCW habitat within 800 m (James et al. 2001) of the territory center to make up the home range size selected. This process led to 144 territories in the baseline landscape; 400 territories in recovery landscape; and 1661 territories in the pre-settlement landscape.

# 6. Input data

**Table A**. Dispersal parameters used to evaluate alternative landscape scenarios. The five models with the minimum –log[Likelihood] for demographic on HS and both demographic and connectivity patterns on CL were selected from POM. The dispersal parameters currently used in the RCW Decision Support System (DSS) were included for comparison.

|  | **pz1** | **pz2** | **pz3** | **pz4** | **pz5** | **DSS** |
| --- | --- | --- | --- | --- | --- | --- |
| **Foray distance of Female Helpers (# cells)** | 51 | 48 | 15 | 60 | 54 | 30 |
| **Foray distance of Female Floaters (# cells)** | 57 | 57 | 57 | 39 | 39 | 30 |
| **Foray distance of Male Helpers (# cells)** | 6 | 3 | 60 | 6 | 3 | 60 |
| **Foray distance of Male Floaters (# cells)** | 54 | 45 | 36 | 54 | 57 | 30 |
| **Female floater and Male natal dispersal speed (# cells)** | 7 | 18 | 20 | 18 | 20 | 6 |
| **Male floater dispersal speed (# cells)** | 18 | 19 | 15 | 18 | 19 | 12 |
| **Male Gap β Terrestrial** | -0.00175 | -0.00175 | -0.00250 | -0.00150 | -0.00130 | 0 |
| **Male Gap β Water** | -0.00300 | -0.00500 | -0.00100 | -0.00375 | -0.00150 | 0 |
| **Female Gap β Terrestrial** | -0.00025 | -0.00250 | -0.00375 | -0.00375 | -0.00250 | -0.00163 |
| **Female Gap β Water** | -0.00425 | -0.00075 | -0.00350 | -0.00275 | -0.00480 | -0.00163 |
| **Female Terrestrial Gap Sensitive Competition (0=off; 1=on)** | 0 | 0 | 0 | 0 | 0 | 1 |
| **Male Terrestrial Gap Sensitive Competition (0=off; 1=on)** | 1 | 0 | 1 | 1 | 0 | 0 |
| **Female Water Gap Sensitive Competition (0=off; 1=on)** | 1 | 1 | 0 | 1 | 1 | 1 |
| **Male Water Gap Sensitive Competition (0=off; 1=on)** | 0 | 0 | 0 | 1 | 1 | 0 |

# 7. Submodels

**Aging**

The age of each bird was incremented one year at the start of every spring. The maximum age recorded for RCW is 17 years (Conner et al., 2001), so birds were not allowed to live past this age.

**Mortality**

Class- and sex- specific mortality rates were derived from from bird banding data (Table B; Walters 2004). We assumed female helpers have the same mortality rates as male helpers.

**Table B. Annual survival probabilities used for the Onslow Bight RCW population**.

| **Class / sex** | **Probability of Annual Mortality** |
| --- | --- |
| Fledgling / male | 0.41 |
| Fledgling / female | 0.56 |
| Helper / male | 0.18 |
| Helper / female | 0.18 |
| Breeder / male | 0.17 |
| Breeder / female | 0.21 |
| Floater / male | 0.30 |
| Floater / female | 0.30 |

**Reproduction**

The number of fledglings produced by each breeding group was estimated based on the influence of demographic and landscape factors on productivity (Table C). We compiled demographic data from 628 breeding events, involving 304 breeding pairs, spanning the years 1997 to 2009 from Marine Corps Base Camp Lejeune. Demographic variables included age of male and female breeder and the number of male helpers in a territory. The number of active territories within 800m of each territory center was also estimated. We first estimate the probability of successful breeding defined as producing at least one fledgling, using logistic regression (i.e., binomial model using a logit link function). In the second step, we estimated the factors that led to *n* fledglings given that the breeding pair was successful. Poisson regression using a log link function was used for this second step. Because both steps involve repeated measures of fledglings generated by a given breeding pair, mixed model regression was used in which breeding pair, a categorical variable, was treated as a random effect and demographic and climatic variables were treated as fixed effects (lmer function in R; R Development Core Team 2010).

**Table C.** Two models were estimated to predict fledglings per group, a logistic and poisson regression, which predict the probability of successful breeding (AIC*c* = 630) and the number of fledglings given at least one fledgling was produced (AIC*c* = 122), respectively.

| **P(Fledglings>0) = logit (p(x))** | | | | |
| --- | --- | --- | --- | --- |
|  | Estimate | Std. Error | *z* value | *P*(>|*z*|) |
| Intercept | -1.80 | 0.469 | -3.85 | 0.000119 |
| age_m | 0.659 | 0.142 | 4.64 | 0.00000345 |
| age_f | 0.578 | 0.124 | 4.67 | 0.00000303 |
| age_f2 | -0.0418 | 0.0101 | -4.14 | 0.0000351 |
| age_m2 | -0.0410 | 0.0103 | -4.00 | 0.0000631 |
| act800 | -0.224 | 0.114 | -1.96 | 0.0500 |
| **Log(E(Fledglings=n|Fledglings>0) )= log(λ(z))** | | | | |
|  | Estimate | Std. Error | *z* value | *P*(>|*z*|) |
| Intercept | 0.596 | 0.0467 | 12.764 | < 2e-16 |
| help_m | 0.122 | 0.0380 | 3.20 | 0.00137 |

Reproduction was modeled by drawing a u[0,1] random numbers and comparing it to the probability of success estimate for that breeding pair. If successful, the Poisson regression equation was used to estimate the number of fledglings. The expected number of fledglings was then used as the estimate of lambda pulled at random from a Poisson distribution.

**Add Immigrants**

This submodel adds one new immigrant that is assumed to be unrelated to all birds included in the simulation. One such unknown immigrant is added every four years, or One Migrant Per Generation.

**Nei**

The expected apportionment of genetic variance within and among breeding groups was estimated by the method of Nei (1973). Total genetic diversity in the population (HT) was apportioned into components including the average genetic diversity within breeding groups (HS) and average genetic divergence among breeding groups (DST­), HT = HS + DST. Nei defines total genetic diversity (HT) as the probability that any two alleles chosen at random are independent. Average gene diversity within breeding groups (HS) equals the probability that two alleles chosen at random from within an individual are independent, averaged across the breeding groups. DST represents the probability that two genes are different when chosen at random from individuals in two different breeding groups (i.e., habitat patches) averaged across the entire population. This method uses allele frequencies of the current population avoiding unrealistic assumptions regarding random uniting of gametes, rates of drift within groups, or patterns of migration among groups (Nei 1986), making it useful for describing genetic diversity in dynamic landscapes.

**Competition**

Competition was simulated largely as described in Letcher et al. (1998). However, a recent radiotelemetry study (Kelser et al. 2010) revealed competition behaviors different than assumptions included in Letcher et al. (1998). It was previously assumed that foray distance, defined as the distance at which a bird can detect and compete for a breeding vacancy, was three km. The recent radiotelemetry results suggest six km as an upper bound on foray distance. We incorporated uncetainty regarding a bird’s foray distance by allowing this value to vary from one to 60 cells, or six km, for helpers and floaters of both sexes. Results from this study also suggested that the presence of non-forested gaps between a bird’s current location and a breeding vacancy may prevent a bird from detecting and competing for that breeding vacancy. We updated the model to included parameters to turn gap avoidance behaviors on or off during competition for breeding vacancies. We included parameters to turn gap avoidance on or off for water and terrestrial gaps during competition for males and females. It was possible for a parameterization to simulate gap avoidance during competition for only terrestrial gaps or both water and terrestrial gaps. If a gap greater than 150 m was present between a competitor’s current location and the breeding vacancy and gap avoidance behaviors were turned on, the individual was unable to compete for the vacancy. However, if gap avoidance was turned off, individuals could compete for any breeding vacancy within its perceptual distance regardless of gaps.

Male-Male Competition

First, we assume that all helpers will preferentially inherit their natal territory upon the death of the breeding male (Walters et al., 1988). If more than one helper is present in a territory when the male breeder dies, we assume the oldest helper wins the territory (Walters et al., 1988). When a helper inherits his natal territory, the female breeder becomes a floater to avoid incest. If the male breeder dies and no helpers are present, it has been observed that 83% of the time the female breeder remains in the territory and acquires a new mate (Daniels and Walters, 2000). It is assumed that widowed females remain in territories until a new male can be established (Letcher et al., 1998). Next competition for widowed female breeders occurs. The model assumes that helpers, male floaters, and solitary male breeders within 3.5 km of the widowed female will compete. The closest male wins the breeding vacancy, if equidistant the oldest male wins (Letcher et al., 1998). Third, competition for vacant territories occurs within 3.5 km of all helpers and male floaters in the area, applying the same rules stated above.

Female-Female Competition

We assume that females only compete for territories containing solitary males and do not defend territories as solitary females (Walters et al., 1988). The pool of female competitors includes all displaced female breeders due to inbreeding avoidance and floaters within 3.5 km of the solitary male. The oldest female wins the vacancy, following Letcher et al. (1998). If there is more than one female of that maximum age, the winner is selected at random. If a female had been a breeder in that territory at a previous time or was born in that territory, she is not allowed to win the territory.

**Natal dispersal**

The probability of a male and female fledgling delaying dispersal and staying as a helper was estimated directly from CL’s bird banding database for the years 1997-2009. For males the probability of dispersal was 0.112 and for females it was 0.629. Following Letcher et al. (1998), each fledgling had a 0.33 probability of dispersing in Summer, Fall, or Winter.

**Dispersal**

Results from the recent radiotelemetry work in the Sandhills Region of NC (Kesler et al. 2010) suggested that birds tend to cross gaps less than 150 m but rarely crossed gaps greater than 600 m, changing their direction of travel to stay within forested areas. A dispersal kernel we refer to as a Gap/Jump dispersal model was derived to capture this dynamic. We estimate the probability of a female crossing gaps > 150 m and < 600 m by:


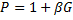


Where, β is a fitted coefficient and G is gap length in meters.

It was assumed that 10% of the time birds would disperse straight through gaps greater than 600 m, in what might be referred to as jumping behavior (Kesler et al. 2010).

POM was used to estimate β for the Onslow Bight landscape within the RCW IB-SEPM. Further, POM allowed us to test new hypotheses, specifically if water gaps affect dispersal behaviors, which is more germane in the Onslow Bight landscape than in the Sandhills. This was accomplished by allowing β to vary independently, from 0 to -0.005, for water and terrestrial gaps for both males and females. We also allowed the number of dispersal steps taken per season to vary, from 1 to 20 cells, or 2 km. If the bird encounters a gap, the dispersal model sums the size of water and terrestrial gaps. For each gap > 150 m and < 600 m the above equation was used to to estimate the probability of crossing the gap. If the gap is greater than 600 m then there was a 10% probability the bird would jump the gap. When each bird encounters a gap the model draws a u[0,1] random number and compares it to the probability of crossing the gap. If the random number is sufficiently small the bird will not cross the gap and turns 45 deg, either right or left chosen at random, to look for a new path. If the bird turns a full 360 deg without being able to move, the bird is left in its current location until the next season at which point it still may be able to compete for a breeding vacancy, may be able to move in the next season, or will die.

**Table D**. The model parameters that were indirectly estimated by POM for males and females and their initial ranges

| **Parameter** | **Range** |
| --- | --- |
| perceptual distance of helpers | 1 to 60 |
| perceptual distance of floaters | 1 to 60 |
| seasonal dispersal distance | 1 to 20 |
| strength of terrestrial gap avoidance | 0 to -0.005 |
| strength of water gap avoidance | 0 to -0.005 |
| sensitivity to terrestrial gaps during competition | 0 or 1 |
| sensitivity to water gaps during competition | 0 or 1 |

# 8. References

Beissinger, S.R., and M.I. Westphal. 1998. On the use of demographic models of population viability in endangered species management. Journal of Wildlife Management 62: 821-841.

Bouzat, J.L. 2001. The importance of control populations for the identification and management of genetic diversity. Genetica 110: 109-115.

Bruggeman, D. J., M. L. Jones, F. Lupi, and K. T. Scribner. 2005. Landscape equivalency analysis: methodology for calculating spatially-explicit biodiversity credits. Environmental Management 36: 518-534.

Bruggeman, D. J., M. L. Jones, K. Scribner, and F. Lupi. 2009. Relating tradable credits for biodiversity to sustainability criteria in a dynamic landscape. Landscape Ecology 24:775–790.

Conner, R.N., D.C. Rudolph, and J.R. Walters. 2001. The Red-cockaded Woodpecker. University of Texas Press, Austin. 363 pp.

Convery, K.M. 2002. Assessing habitat quality for the endangered Red-Cockaded Woodpecker (*Picoides borealis*). Master’s Thesis submitted to the Faculty of Virginia Polytechnic Institute and State University, Blacksburg VA.

Daniels, S. J., J. R. Walters. 2000. Inbreeding depression and its effects on natal dispersal in red-cockaded woodpeckers. Condor 102: 482-491

deGroot, R.S., M.A. Wilson, and R.M.J. Boumans. 2002. A typology for the classification, description and valuation of ecosystem functions, goods, and services. Ecological Economics 41: 393-408.

Franzreb, Kathleen E., 2006. Implications of home-range estimation in management of red-cockaded woodpecker in South Carolina. Forest Ecology and Management 228: 274-284.

Grimm V, U. Berger, D.L. DeAngelis, G. Polhill, J. Giske, S.F. Railsback. 2010. The ODD protocol: a review and first update. Ecological Modelling 221: 2760-2768

Grimm, V., E. Revilla, U. Berger, F. Jeltsch, W. Mooij, S.F. Railsback, H.H. Thulke, J. Weiner, T. Wiegand, and D.L. DeAngelis. 2005. Pattern-oriented modeling of agent-based complex systems: lessons from ecology. Science 310: 987-991.

James F. C., C. A. Hess, B. C. Kicklighter, and R. A. Thum. 2001. Ecosystem management and the niche gestalt of the red-cockaded woodpecker in longleaf pine forests. Ecological Applications 11:854-870.

Jones, C.A., and K.A. Pease. 1997. Restoration-based compensation measures in natural resource liability statutes. Contemporary Economic Policy 15: 111-122.

Kesler, D.C, J.R Walters, and J.J. Kappes. 2010. Social influences on dispersal and the fat-tailed dispersal distribution in red-cockaded woodpeckers. Behavioral Ecology 21: 1337-1343.

Lacy, R.C., and D.B. Lindenmayer. 1995. A simulation study of the impacts of population subdivision on the mountain brushtail possum *Trichosurus caninus* Ogilby (Phalangeridae: Marsupialia), in south-eastern Australia. II. Loss of genetic variance within and between subpopulations. Biological Conservation 73: 131-142.

Letcher B. H., J. A. Priddy, J. R. Walters, and L. B. Crowder. 1998. An individual-based, spatially-explicit simulation model of the population dynamics of the endangered red-cockaded woodpecker, Picoides borealis. Biological Conservation 86:1-14.

Loomis, J.B., and D.S. White. 1996. Economic benefits of rare and endangered species: summary and meta-analysis. Ecological Economics 18: 197-206.

Manel S, MK Schwartz, G Luikart, P Taberlet (2003) Landscape genetics: combining landscape ecology and population genetics. *Trends in Ecology and Evolution,* **18,** 189-197.

McDearman, W. 2011. Southern Range Translocation Cooperative: red-cockaded woodpecker (Picoides borealis) translocation success 2007-2009. Unpublished file report. U.S. Fish and Wildlife Service, Atlanta, GA.

Meffe, G.K. 1996. Conserving genetic diversity in natural systems. Pages 41-57 in R.C. Szaro and D.W. Johnston (eds.) Biodiversity on managed landscapes: theory and practice. Oxford University Press, New York.

Mills, L.S., and F.W. Allendorf. 1996. The one-migrant-per-generation rule in conservation and management. Conservation Biology 10: 1509-1518.

Moritz, C. 2002. Strategies to protect biological diversity and the evolutionary processes that sustain it. Systematic Biology 51: 238-254.

Nei M (1986) Definition and estimation of fixation indices. Evolution 40: 643-645.

Nei, M. 1973. Analysis of gene diversity in subdivided populations. Proceedings of the National Academy of Science, USA 70: 3321-3323.

R Core Development Team. 2010. R: A language and environment for statistical computing. R Foundation for Statistical Computing: Vienna, Austria. <http://www.r-project.org/>

U.S. Fish and Wildlife Service. Recovery plan for the red-cockaded woodpecker (*Picoides borealis*): second revision. Atlanta, GA: U.S. Fish and Wildlife Service; 2003.

Walters J. R., S. J. Daniels, J. H. Carter III, and P. D. Doerr. 2002. Defining Quality of Red-Cockaded Woodpecker Foraging Habitat Based on Habitat Use and Fitness. The Journal of Wildlife Management 66:1064-1082.

Walters, J. R. 2004. Unusual dynamics in a rapidly increasing population of Red-Cockaded Woodpeckers at Camp Lejeune, North Carolina. Pages 256-267 *in* R. Costa and S. J. Daniels, eds. Red-Cockaded Woodpecker: Road to Recovery. Hancock House Publishing, Blaine, WA.

Walters, J.R., P.D. Doerr, and J.H. Carter, III. 1988. The cooperative breeding system of the red-cockaded woodpecker. Ethology 78: 275-305.

Wang J (2004) Application of the one-migrant-per-generation rule to conservation and management. *Conservation Biology* **18**, 332-343.

Wood, D. R., Vilella, F. J., Burger, L. W. 2008. Red-cockaded woodpecker home range use and macrohabitat selection in a loblolly-shortleaf pine forest. The Wilson journal of Ornithology. 120 (4); 793-800.

Wright S (1969) Evolution and the genetics of populations. Vol. 2. *The theory of gene frequencies*. University of Chicago Press.
