# Supplementary material for: The Value of Learning about Natural History in Biodiversity Markets
Source: PLoS One. 2015 Dec 16;10(12):e0144047. doi: 10.1371/journal.pone.0144047 (PMC4684417; doi:10.1371/journal.pone.0144047)
Supplement: S1 Table — (DOC) [file pone.0144047.s002.doc]

| **S1 Table. Estimated costs and profits for the hypothetical RCW conservation bank with 25 territories.** | | |
| --- | --- | --- |
|  | Estimate | Source |
| Fee simple purchase ($10k/ha) | $18,250,000 | Approximate cost based on review of Zillow.com and discussions with longleaf pine managers |
| Initial mechanical understory clearing | $456,250 | RCW Recovery Plan [21] |
| Installation of 4 cavities for each of the 25 RCW territories | $20,000 | RCW Recovery Plan [21] |
| Prescribed burns every 3 years for the 15 years of habitat maturation | $456,250 | RCW Recovery Plan [21] |
| Costs on a yearly-basis including bird monitoring & prescribed burning (every 3-years) | $70,417 | RCW Recovery Plan [21] & Past field efforts by the author |
| Non-wasting endowment | $1,500,000 | Estimated to cover burning and monitoring costs while allowing sufficient re-investment to off-set inflation |
| 8% Profit | $1,660,233 |  |
|  |  |  |
| Total Costs & Profit | $22,342,733 |  |
